# A novel inhibitor of the jasmonic acid signaling pathway represses herbivore resistance in tea plants

**DOI:** 10.1093/hr/uhab038

**Published:** 2022-01-19

**Authors:** Songbo Lin, Meng Ye, Xiwang Li, Yuxian Xing, Miaomiao Liu, Jin Zhang, Xiaoling Sun

**Affiliations:** 1 Tea Research Institute, Chinese Academy of Agricultural Sciences, No. 9 South Meiling Road, Hangzhou 310008, Zhejiang, China; 2 Key Laboratory of Tea Biology and Resources Utilization, Ministry of Agriculture and Rural Affairs, No. 9 South Meiling Road, Hangzhou 310008, Zhejiang, China

## Abstract

The jasmonic acid (JA) signaling pathway plays a vital role in mediating plant resistance to herbivores*.* The tea plant (*Camellia sinensis*) is one of the most important woody cash crops in the world. Due to the lack of genetic transformation systems for tea plants, how the JA signaling pathway works in tea plants has not yet been determined. Now, with the development of cross-disciplines, chemical biology provides new means for analyzing the JA signaling pathway. In the present study, the structure of the small-molecule isoquinoline compound ZINC71820901 (lyn3) was obtained from the ZINC molecular library through virtual screening based on the structure of the crystal COI1-JAZ1 co-receptor and was found to act as an inhibitor of the JA signaling pathway in both *Arabidopsis* and tea plants. Our results revealed that lyn3 repressed tea plant resistance to *Ectropis grisescens* mainly by decreasing the accumulation of (−)-epicatechin and (−)-epigallocatechin via repression of the JA signaling pathway, which functioned in a modulation manner different from that of the already known inhibitor salicylhydroxamic acid (SHAM). As a novel inhibitor of the JA signaling pathway, lyn3 provides a specific option for further research on the JA pathway.

## Introduction

In response to being challenged by a multitude of herbivores, sessile plants have evolved complicated and sophisticated resistance systems [1–3], including constitutive physical defenses, such as trichomes, to obstruct herbivore feeding; induced direct defenses, such as antinutrient proteins and toxic secondary metabolites, to impede pest growth; and induced indirect defenses, such as herbivore-induced plant volatiles (HIPVs), to recruit beneficial organisms or prime the defense of neighboring plants [4], all of which are modulated by multiple phytohormones, including jasmonic acid (JA), salicylic acid (SA), and ethylene [5]. Among all the defense hormones, the lipid-derived plant hormone JA and its derivatives play a central role [2, 6, 7]. Herbivore attack or mechanical wounding alone triggers the biosynthesis of JA in plants from α-linolenic acid through a series of synthases, including 13-lipoxygenase (13-LOX) [8], allene oxide synthase (AOS) [9], allene oxide cyclase (AOC) [10], and OPDA reductase 3 (OPR3) [11]. Subsequently, jasmonate-amido synthetase 1 (JAR1) conjugates JA to isoleucine (Ile), generating a biologically highly active enantiomer of (+)-7-iso-JA-L-Ile (JA-Ile) [12], which gives rise to an interaction between the receptor F-box protein COI1 and repressor family JAZs (jasmonate-ZIM domains) [13], resulting in the large-scale transcriptional reprogramming of many transcription factors, such as *MYCs* [14], *MYBs* [15], and a large number of JA-responsive genes [16], which trigger a series of JA-dependent downstream responses, such as the biosynthesis of defensive proteins [thionin (encoded by *THI*) and vegetative storage protein 1 (encoded by *VSP1*)] and several metabolites [14].

Although plant transgenic technology has led to great advances in recent decades in research on the JA signaling pathway in model plants by using corresponding mutants [2, 7], the genetic redundancies and shortcomings of valid genetic transformation systems for most non-model plants limit deeper investigation, especially woody plants [17]. With the development of cross-disciplines, chemical biology provides new means for analyzing the JA signaling pathway [18]. The bacterial toxin coronatine has been found to be a structural mimic of JA-Ile with enhanced bioactivity [19]. However, it has been shown that random chemical modifications of JA and coronatine usually decrease their efficiency [20–22]. With further research on the JA signaling pathway, especially confirmation of the perception mode of JA-Ile with the COI1-JAZ co-receptor [23], rational design of the regulator in the JA signaling pathway has become possible. For instance, coronatine-*O*-methyloxime (COR-MO) prevents the COI1-JAZ interaction through a methyl oxime group blocking the keto group that interacts with JAZ proteins [24]; NOPh, a phenyloxime derivative based on the coronatine stereoisomer, selectively interacts with COI1 and different JAZs, leading to the uncoupling of pathogen resistance and inhibiting the growth of *Arabidopsis* [25]. In recent years, computer-aided design has been increasingly utilized in the discovery of novel regulators of the JA signaling pathway. ZINC27640214 and ZINC43772052 are two analogues of JA identified by virtual screening and molecular dynamics simulations based on the structure of COI1, which has a stronger binding affinity to COI1 than JA [26]. Unfortunately, the structure of COI1 used to identify these two JA analogues was not a crystal structure and the function of JAZ was ignored; therefore, further study is needed to gain a better understanding of the JA signaling pathway. Moreover, most of the above-mentioned chemicals were designed to regulate defense and resistance to pathogens in model plants. Except for some already reported regulators of the JA signaling pathway, such as jasmonate elicitors and two inhibitors [sodium diethyldithiocarbamate trihydrate (DIECA) and salicylhydroxamic acid (SHAM)], few new regulators that mediate plant resistance to herbivores in non-model plants have been identified so far.

The tea plant *Camellia sinensis* is one of the most important woody cash crops in the world, and usually suffers attack from numerous pests, especially the tea geometrid (*Ectropis obliqua* Prout) and its sibling species (*E. grisescens* Warren) (Supplementary Fig. S1). Transcriptome analysis and qRT–PCR results have demonstrated that *E. obliqua* attack triggers the expression of JA biosynthesis-related genes and transcription factors [27]. Additionally, more robust evidence has illustrated that the JA signaling pathway plays a pivotal role in the regulation of tea plant resistance to *E. obliqua* [28, 29]. For example, the JA content has been shown to be increased significantly upon attack by *E. obliqua*, and the weight gain changed in opposite directions when comparing geometrids fed on plants treated with JA versus SHAM [28]. Although a series of JA-related genes and their expression patterns have been reported in tea plants, such as *CsACX, CsOPR3*, and *CsLOX*, there have been no effective methods for determining how the JA signaling pathway works in tea plants until now, which has greatly hindered progress in improving the herbivore resistance of tea [30–32]. Therefore, the design, screening, and identification of small molecular chemicals that can modulate tea plant resistance against tea pests or pathogens is urgent and would be helpful in regulating tea resistance when needed.

The objectives of this study were to uncover a modulator of the JA signaling pathway based on the crystal COI1-JAZ1 co-receptor from the ZINC molecular library through virtual screening and then to assess the impact of the modulator on tea plant resistance to *E. grisescens* caterpillars [33]. The impacts of the modulator on the production of defense-related genes, phytohormones, and secondary metabolites were assessed, and the antifeeding effects of different key compounds on *E. grisescens* caterpillars were measured by using an artificial diet supplemented with them. Finally, the elicitor JA was used exogenously to verify the steering function of lyn3. Our work designed a novel inhibitor of the JA signaling pathway, which provides a specific option for research on the JA signaling pathway in tea plants.

## Results

### Virtual screening

**Figure 1:**
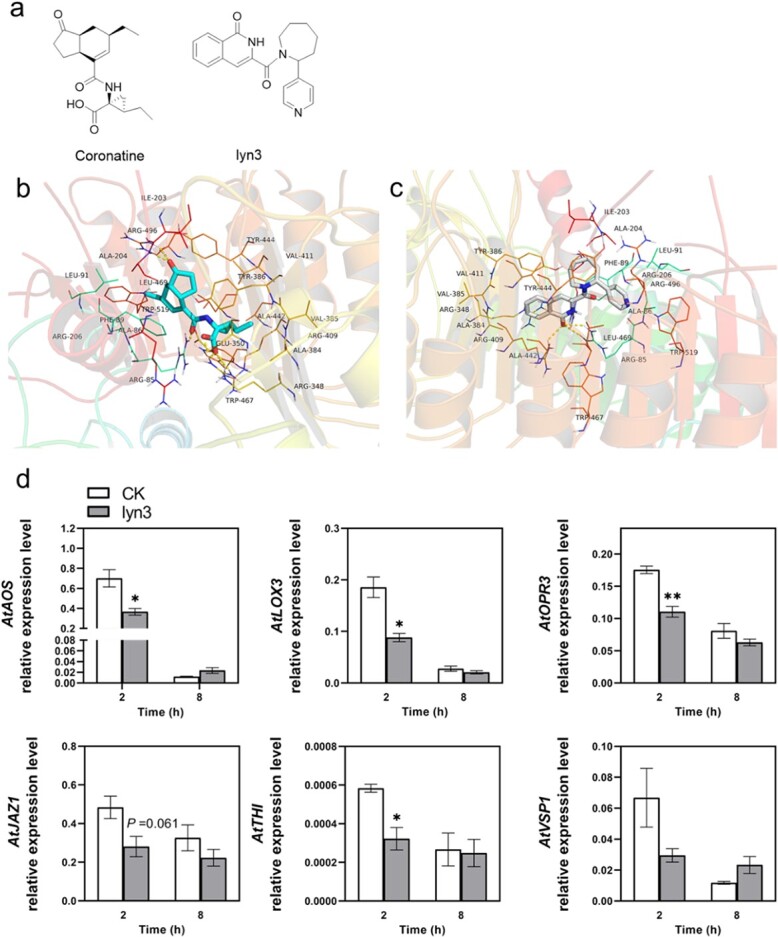
Lyn3 steers the JA signaling pathway in *Arabidopsis*. **a** Chemical structures of coronatine and lyn3. **b** and **c** Side views of coronatine and lyn3 binding in the AtCOI1–JAZ1 pocket; backgrounds are cartoons of the structure of AtCOI1–JAZ1 complex at different angles. **d** Exogenous application of lyn3 suppresses expression levels of JA-related genes in *Arabidopsis* (mean ± standard error, *n* = 3). Asterisks indicate significant differences between treatment and control (CK): ^*^P < .05, ^**^P < .01, Student’s *t*-test.

To obtain lead compounds for chemical regulators of the JA signaling pathway, the COI1-JAZ1 protein complex downloaded from the RCSB Protein Data Bank (PDB) was used as a receptor to virtually screen ~6 000 000 molecules from the ZINC lead-like library using AutoDock Vina. The binding energy score of ZINC71820901 was −11.5 kcal/mol, much lower than the −10.6 kcal/mol of coronatine. After visual analysis of the binding modes of ZINC71820901 (Fig. 1a), which is also named lyn3, we found that lyn3 bound tightly with the COI1 protein. Deeper in the binding pocket, the keto group of isoquinolin-1(2H)-one formed a triangular hydrogen bond network with Arg 85 and Arg 409 of COI1. The azepane group was stabilized by hydrophobic packing by the aromatic groups of Phe 89 and Tyr 444. Meanwhile, the benzene ring of the isoquinolinone in lyn3 paralleled well with the phenyl group of Try 386, forming a π–π stacking interaction (Fig. 1c). However, no obvious contacts were observed between lyn3 and JAZ1, which indicated that lyn3 might occupy the cavity of COI1, interfering with interactions between COI1 and JAZs.

### Lyn3 treatment suppresses the expression levels of JA-related genes in *Arabidopsis*

To explore the influence of lyn3 on the JA signaling pathway, the relative expression levels of several JA-related genes in *Arabidopsis* were quantified. As shown in Fig. 1d, the expression levels of *AtAOS*, *AtLOX3*, *AtOPR3*, *AtTHI*, and *AtJAZ1* were significantly suppressed, by 48, 53, 37, 45, and 42%, respectively, compared with that of the control at 2 h after the start of treatment. However, there was no significant difference between treatment and control at 8 h.

### Lyn3 treatment suppresses herbivore resistance in tea plants

**Figure 2:**
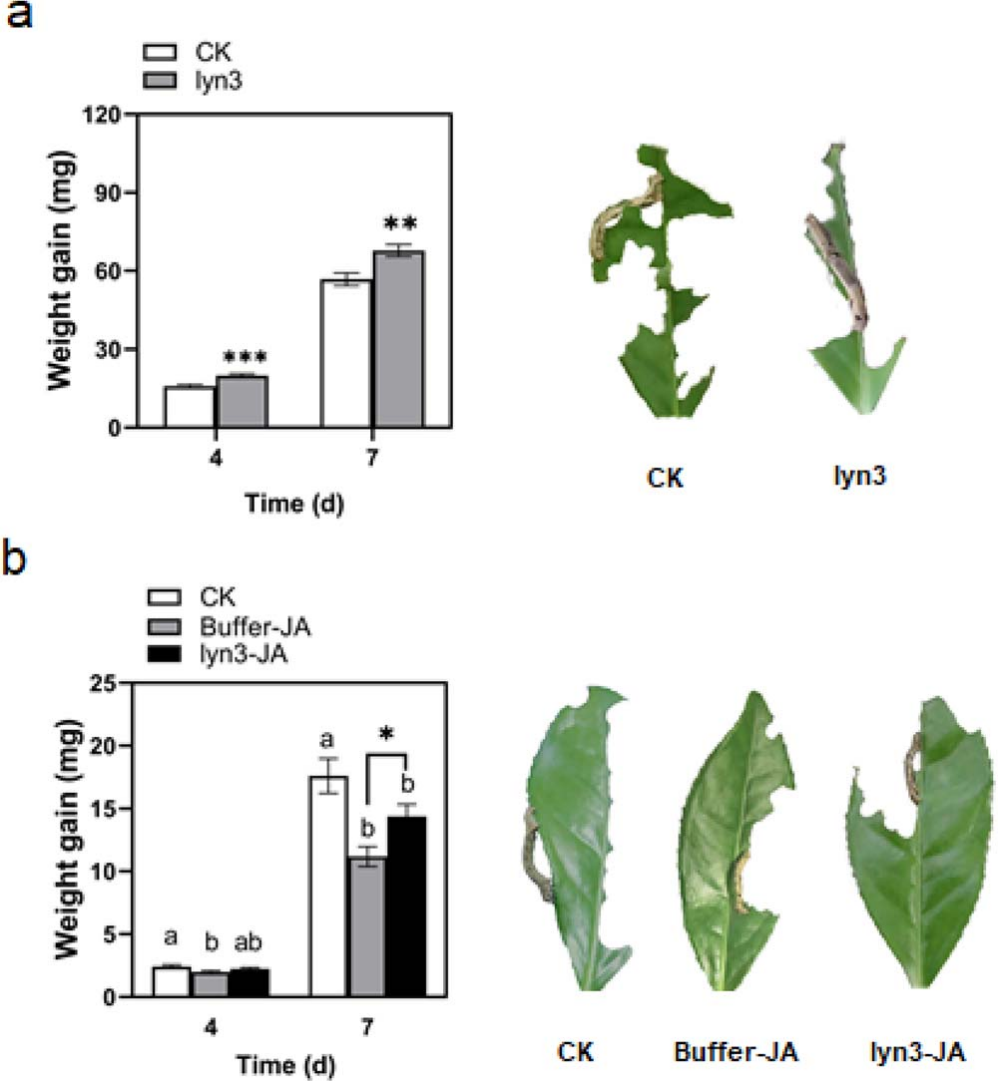
Effect of lyn3-treated (**a**) and lyn3-JA-teated (**b**) tea plants on *E. grisescens* larval weight gain (mean ± standard error, *n* = 51–68). Buffer is composed of 0.15% 1 M hydrochloric acid, 0.1% ethanol, and 0.1% Tween 20. Different letters indicate significant differences among treatments: *P* < .05, Tukey’s HSD *post hoc* test. Asterisks indicate significant differences between treatment and control (CK): ^*^P < .05, ^**^P < .01, ^***^P < .001, Student’s *t*-test.

The weight gain of *E. grisescens* larvae fed on lyn3-treated tea plants was significantly higher than that of larvae fed on control plants. Four and 7 days after the start of feeding, the weight gains of *E. grisescens* larvae fed on lyn3-treated plants were 26 and 19% higher than that of those fed on control plants, respectively (Fig. 2a). To make sure that the exogenous application of lyn3 mediated tea plant resistance through the JA signaling pathway, lyn3 and JA were applied to tea leaves successively, and the weight gain of *E. grisescens* larvae fed on the treated leaves was recorded after 4 and 7 days. As shown in Fig. 2b, the weight gains of *E. grisescens* larvae fed on JA-treated leaves and lyn3 plus JA-treated leaves were significantly lower than those of larvae fed on control leaves at 7 days. However, the weight gain of *E. grisescens* caterpillars fed on lyn3 plus JA-treated tea leaves was significantly higher than that of those fed on buffer plus JA-treated leaves at 7 days. Furthermore, we found that *E. grisescens* larvae fed on an artificial diet supplemented with lyn3 at the corresponding concentration grew more slowly than those fed on a control artificial diet, which indicated the direct antifeeding effect of lyn3 on *E. grisescens* larvae (Supplementary Fig. S2). In summary, lyn3 pretreatment suppresses herbivore resistance in tea plants through interfering with plant metabolism rather than by a direct effect of lyn3.

### Lyn3 treatment represses induced JA content in tea plants

**Figure 3:**
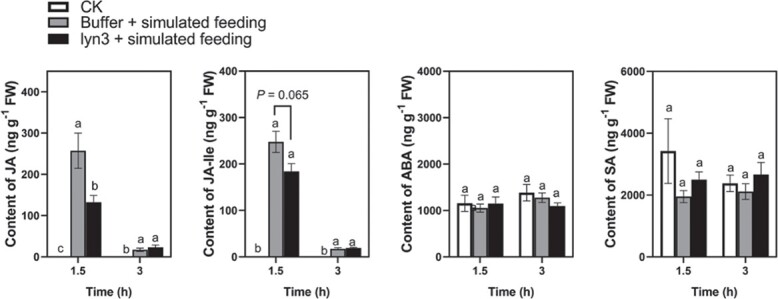
Effect of lyn3 on the contents of phytohormones (mean ± standard error, *n* = 4). Buffer is an aqueous solution containing 0.15% 1 M hydrochloric acid, 0.1% ethanol, and 0.1% Tween 20. Different letters indicate significant differences among treatments: *P* < .05, *n* = 4, Tukey’s HSD *post hoc* test.

Compared with buffer-treated plants, simulated *E. grisescens* feeding significantly increased the contents of JA and JA-Ile at 1.5 and 3 hours after the start of treatment. Compared with simulated feeding alone, pretreatment with lyn3 followed by feeding decreased the contents of JA and JA-Ile by 48 and 26%, respectively, at 1.5 hours after the start of feeding. There were no significant differences in the contents of SA and abscisic acid (ABA) among the three treatments at each time point (Fig. 3).

### Lyn3 treatment represses the flavonoid pathway in tea plants

**Figure 4:**
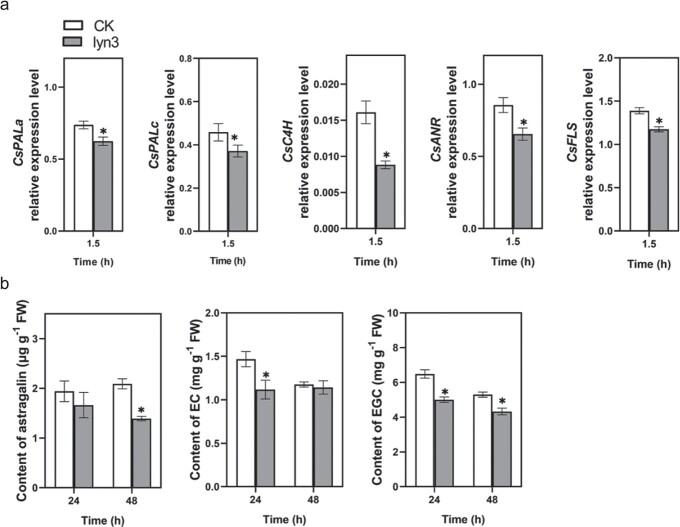
Lyn3 treatment suppresses the flavonoid pathway in tea plants. **a** Relative expression levels of five genes in the flavonoid pathway (mean ± standard error, *n* = 4). **b** Accumulation of flavonoids was repressed significantly by lyn3 treatment (mean ± standard error, *n* = 4). The asterisk indicates a significant difference between treatment and control (CK): ^*^P < .05, Student’s t-test.

According to previous reports, the expression levels of five genes [phenylalanine ammonia-lyase (*PAL*), *trans*-cinnamate 4-monooxygenase (*C4H*), anthocyanidin reductase (*ANR*) and flavonol synthase (*FLS*)] related to flavonoid biosynthesis were measured. The results showed that the expression levels of most of these genes were reduced by lyn3 treatment. Compared with those of the control, the expression levels of *CsPALa*, *CsPALc*, *CsC4H*, *CsANR*, and *CsFLS* were significantly decreased by 15, 19, 45, 23, and 15% at 1.5 hours after treatment with lyn3 (Fig. 4a).

Considering the downregulation of the transcriptional expression levels of flavonoid biosynthesis genes steered by lyn3, the contents of a total of 18 flavonoids in tea plants were assessed (Supplementary Fig. S3). Compared with the control, the accumulation of three compounds in lyn3-treated tea leaves was decreased (Fig. 4b). In detail, the contents of (−)-epicatechin (EC) and (−)-epigallocatechin (EGC) in lyn3-treated tea leaves were 24 and 23% lower than those in control leaves, respectively, at 24 hours after the start of treatment; the contents of astragalin and EGC in lyn3-treated tea leaves were reduced by 33 and 18%, respectively, at 48 hours after the start of treatment. Although the accumulations of catechin, epigallocatechin, gallocatechin gallate, and neoschaftoside in tea leaves were slightly repressed by lyn3 treatment, there was no significant difference between treatment and control (Supplementary Fig. S3).

### Antifeeding effects of differential flavonoids on herbivore performance

**Figure 5:**
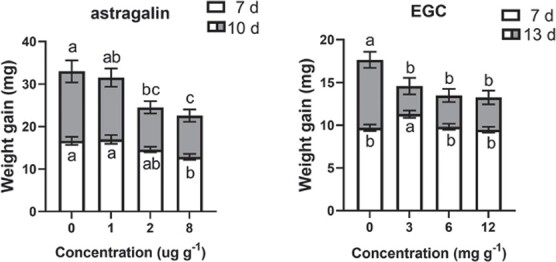
Effects of an artificial diet supplemented with differential substances on *E. grisescens* larval weight gain. Data are mean weight (± standard error), *n* = 43–54. Different letters indicate significant differences among treatments: *P* < .05, Tukey’s HSD *post hoc* test.

The antifeeding effect of EC on *E. grisescens* performance was described in our other report [34]. Only astragalin and EGC were tested in the present study. The results are shown in Fig. 5. The effect of astragalin on the weight gain of *E. grisescens* larvae was dependent on the concentration of astragalin (*F*_3,527_ = 16.512, *P <* .001) and feeding time (*F*_1,527_ = 136.318, *P <* .001). Generally, the higher the concentration of astragalin in the diet, the lower the larval mass, and the effect intensified as the feeding time increased. A negative correlation was found between the astragalin level and larval mass at 7 days after feeding (*r* = −.202, *P* = .001) and 10 days after feeding (*r* = −.250, *P* = .003). Seven days after the start of feeding, the weight gain of larvae fed on an artificial diet supplemented with 8 μg g^−1^ astragalin was reduced by 23% compared with that of larvae fed the control diet. Ten days after the start of feeding, the weight gain of larvae fed on an artificial diet supplemented with 2 and 8 μg g^−1^ astragalin was reduced by 27 and 32% compared with larvae fed on a control diet, respectively.

The effect of EGC on larval weight gain was dependent on the concentration of EGC (*F*_3,358_ = 4.788, *P <* .001) and feeding time (*F*_1,360_ = 99.447, *P <* .001). There was no correlation between the concentration of EGC and larval weight gain at 7 days after feeding, while at 13 days after feeding a correlation was found (*r* = −.253, *P* = .001), Thirteen days after the start of feeding, the weight gain of larvae fed on an artificial diet supplemented with 3, 6 and 12 mg g^−1^ EGC was reduced by 15, 22, and 23%, respectively, compared with that of larvae fed on the control diet (Fig. 5).

### The JA signaling pathway modulates biosynthesis of flavonoids

**Figure 6:**
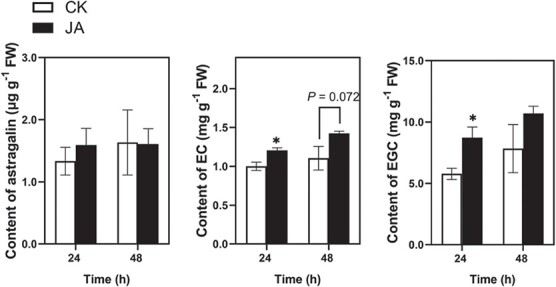
Contents of differential flavonoids elicited in tea plants by exogenous application of activator JA (mean ± standard error, *n* = 5). Asterisks indicate significant differences between two treatments: ^*^P < .05, Student’s *t*-test. CK, control.

To confirm the modulatory effect of the JA signaling pathway on the biosynthesis of flavonoids in tea plants, the elicitor JA was used. Exogenous application of JA significantly induced the accumulation of EC and EGC but had no effect on the accumulation of astragalin (Fig. 6). Except for the above flavonoids, the contents of apigenin 5-*O*-glucoside and neoschaftoside in JA-treated tea leaves were 44 and 33% lower than those in control leaves, respectively, at 24 h after the start of treatment, while exogenous application of JA significantly elicited the accumulation of naringenin and slightly, but not significantly, elicited the accumulations of the other 12 flavonoids (Supplementary Fig. S6).

## Discussion

JA has been identified as a signaling molecule that mediates a variety of biological responses in plants, including plant growth, male sterility, the production of secondary metabolites, and defense against attack by herbivorous insects or necrotrophic pathogens [35]. The application of mutant plants has greatly advanced research on JA biosynthesis and signal transduction [7]. However, the significant genetic redundancy of genes related to the JA signaling pathway and the deficiency of the genetic transformation systems for most non-model plants have greatly hampered advances in identifying genes that encode enzymes with the physiological functions of the JA pathway in these plants, for which chemical biology provides an alternative option [18, 36]. The vastly improved understanding of the COI1–JAZ co-receptor system has enabled the rational design of JA antagonists and agonists, which makes it possible to further probe the JA pathway [23, 37]. For example, based on the binding mode of coronatine to the COI1-JAZ co-receptor, the specific antagonist COR-MO was designed, which interfered with JA-Ile perception by blocking the keto residue [24]. However, the raw material required to produce COR-MO is expensive, and the oximido is unstable under acidic conditions, which hinders the application of this antagonist in agricultural production. ZINC27640214 and ZINC43772052 were obtained through the virtual screening of 767 analogues of JA from the ZINC database based on the high-quality structural model of COI1 [34]. But the structure of COI1 used to identify these two compounds was not a crystal structure, and the function of JAZ was ignored. Here, we screened >6 000 000 million molecules from the ZINC lead-like molecular library based on an accurate structure of *Arabidopsis* COI1-JAZ. Considering its binding energy and synthetic availability, we selected ZINC71820901 as the lead compound for the JA regulator and named it lyn3. After visual analysis of the docking results of lyn3 with the COI1-JAZ co-receptor, we found that the high binding energy of lyn3 with COI1-JAZ might mainly be attributed to hydrogen bonds and π–π stacking with COI1, and there was no interaction with the JAZ repressor (Fig. 1c), which indicated that lyn3 might have a similar function to COR-MO as a JA antagonist. Consistent with our hypothesis, exogenous application of lyn3 significantly suppressed the expression of JA-responsive genes in *Arabidopsis*, such as *AtAOS*, *AtLOX3*, *AtOPR3*, *AtTHI*, and *AtJAZ1* (Fig. 1d), but how lyn3 works in non-model plants, such as tea plants, requires further investigation. Because lyn3 was obtained from a virtual screening based on the binding mode of JA-Ile with *Arabidopsis* COI1-JAZ, we firstly performed yeast two-hybrid assays to investigate the inhibitory effect of lyn3 on the protein–protein interaction of COI1-JAZ in tea plants caused by coronatine. Unexpectedly, the results showed that lyn3 had no influence on the interaction (Fig. S4), which suggests that virtual screening can be used as a tool to identify potential active compounds, but their actual functions and underlying mechanisms in non-model plants need to be further investigated.

JA plays a vital role in mediating herbivore resistance in several plants [35]. Several lines of evidence have demonstrated that the JA signaling pathway mediates tea plant resistance to tea geometrids. First, the JA level was significantly increased by the infestation of *E. grisescens* larvae [38]. Second, the induction of direct resistance of tea plants via increasing polyphenol oxidase activity was demonstrated to depend on the JA signaling pathway by exogenous application of JA and SHAM following by *E. grisescens* feeding [29, 39]. Third, treatment with the green leaf volatile (*Z*)-3-hexenol increased constitutive and *E. grisescens*-induced JA levels in tea plants, resulting in the enhancement of direct and indirect plant defenses against tea geometrids [40]. Fourth, treatment with the jasmonate derivative JA-Ile-macrolactone 5b significantly increased the defensive capacity of tea plants against *E. grisescens* [41]. Finally, indole exposure increased tea plant resistance to *E. grisescens* by boosting Ca^2+^ and JA signaling, resulting in enhanced jasmonate-dependent defense and resistance [42]. Due to the lack of genetic transformation systems for tea plants, it is important and urgent to explore novel regulators of the JA signaling pathway. Previously, we found that SHAM treatment increased the performance of *E. grisescens* and reduced the *E. grisescens*-elicited expression levels of the lipoxygenase gene *CsLOX1* and the putative allene oxide synthase gene *CsAOS1*, suggesting that SHAM negatively mediates herbivore-induced defense by blocking the biosynthesis of JA in tea plants [28]. In the current study, the weight gain of *E. grisescens* fed on lyn3-treated tea plants was significantly higher than that of those fed on control plants (Fig. 2a). More importantly, we found that lyn3 suppressed JA-induced herbivore resistance in tea plants (Fig. 2b). Furthermore, we found that lyn3 treatment decreased the contents of JA and JA-Ile induced by *E. grisescens* simulated feeding but had no significant influence on the contents of ABA and SA (Fig. 3). In contrast to SHAM, exogenous application of lyn3 had no significant influence on the expression of JA-related genes elicited by *E. grisescens* simulated feeding in tea plants (Fig. S5). Taken together, these results suggested that lyn3 negatively mediated the resistance of tea plants to *E. grisescens* by inhibiting the JA signaling pathway in a different manner from SHAM, but the detailed mechanism needs to be further explored.

Flavonoids, including aurones, flavans, flavones, flavonols, proanthocyanidins, dihydroflavonols, flavanones, chalcones, and anthocyanins, have been shown to play central roles in plant–environment interactions [43]. Metabolome results have shown that *E. grisescens* infestation elicits the accumulation of flavonoids in both resistant and susceptible tea cultivars [44]. However, tea green leafhopper (*Empoasca onukii* Matsuda) attack induced the formation of theaflavins from catechins under the action of polyphenol oxidase [38]. In addition, epigallocatechin and catechin were found to be involved in the resistance of tea plants to the tea anthracnose *Colletotrichum camelliae* [45]. In the present study, lyn3 treatment downregulated the expression levels of several genes related to flavonoid biosynthesis in a tea plant (Fig. 4a), leading to a reduction in the accumulation of astragalin, EC, and EGC (Fig. 4b). In the current study, astragalin and EGC exhibited an antifeeding effect on *E. grisescens* caterpillars (Fig. 5), while EC significantly inhibited the growth rate of *E. grisescens* caterpillars according to our study [34]. Further research revealed that JA treatment induced the accumulation of EC and EGC in tea plants (Fig. 6). Taken together, our results demonstrate that lyn3 reduced tea plant resistance to *E. grisescens* mainly by decreasing the accumulation of EC and EGC via repression of the JA signaling pathway. It is worth noting that our study specifically focuses on the flavonoid biosynthesis pathway and reveals the antifeeding compounds based on this; other metabolic pathways involving in this process need to be investigated in the future experiments.

In conclusion, the small-molecule isoquinoline compound lyn3 screened from the ZINC molecular library was obtained through virtual screening based on the structure of the COI1-JAZ1 protein complex and was found to act as a novel inhibitor of the JA signaling pathway. Our results revealed that lyn3 repressed tea plant resistance to *E. grisescens* mainly by decreasing the accumulation of EC and EGC via repression of the JA signaling pathway, which functioned in a modulation manner different from that of the already known inhibitor SHAM. As a novel inhibitor of JA, lyn3 provides a specific option for further research on the JA pathway. Furthermore, by studying the lyn3 binding mode with COI1-JAZ, novel antagonists or agonists can be rationally designed.

## Materials and methods

### Virtual screening based on the structure of COI-JAZ1 protein complex

The crystal structure of COI-JAZ1 protein [Protein Data Bank (PDB) ID: 3OGK] was downloaded from the RCSB PDB. H_2_O, PO_4_ and coronatine were deleted from the crystal structure in AutoDock tools [33]. Then polar hydrogen atoms were added and Gasteiger charges were adjusted. Finally, the macromolecule was saved as receptor.pdbqt. The grid box was selected and optimized based on the location and size of the original ligand, coronatine [45]. The coordinates of the center of the binding site were assigned as 0.500, 4.583, 47.889. The size of the grid box was selected as 30 × 30 × 26 Å. The lead-like molecular library was downloaded from ZINC [47], then the file was decompressed and all the molecules were transformed to pdbqt files via the Raccoon program. This was performed automatically for each molecule by docking the ligand to the receptor one by one in the AutoDock Vina program via the Python language at the Linux-based server (Dell Inc., TX, USA). After molecular docking, the binding affinity scores were extracted through Python and ranked in Excel. The top 60 molecules, whose affinity scores were <−11.5 kcal/mol, were selected and the docking results were extracted and analyzed via pyMOL (Schrodinger LLC, New York, USA).

### Chemical reagents

Lyn3 (ZINC71820901) was purchased from Enamine (Kievska, Ukraine), and was dissolved in 0.15 mL of 1 M HCl and 0.1 mL of ethanol, then diluted with 0.1% Tween 20 aqueous solution to the concentrations of 1.0 mM and 1 μM separately. A solution containing the same amounts of HCl, ethanol and Tween 20 was used as the control buffer. Flavonoids were purchased from Yuanye Bio-Technology Co., Ltd (Shanghai, China); JA and SHAM were purchased from J&K Chemical Ltd (Shanghai, China).

### Plant cultivation

*Arabidopsis* (Col-0) seeds were sterilized with sodium hypochlorite solution, washed with sterile water, and chilled at 4°C for 3 days, plated on the Murashige and Skoog medium, then transferred to plant incubator under a photoperiod of 14:10 h (light:dark) at 23 ± 2°C and 60% relative humidity.

Three-year-old *C. sinensis* (L.) O. Kuntze ‘Longjing43’ tea plants, which were planted individually in plastic pots, were transported to a walk-in climate chamber (25 ± 2°C, relative humidity 70–80%, light:dark 14:10 h) and kept for at least 3 days before treatment.

### Insects

*E. grisescens* eggs were collected from mated female adults, which were reared continuously in a controlled climate room (26 ± 2°C, relative humidity 70 ± 5%, light:dark 12:12 h). Fourth-instar caterpillars were used for regurgitant collection. Three-day-old healthy caterpillars of the same size were selected for the bioassays.

### Plant treatments

#### Arabidopsis

Seven-day *Arabidopsis* seedlings were soaked with 1 μM lyn3 or buffer solution separately for 2 and 8 h, and were used as treatment and control. Ten seedlings were pooled into one sample. Each treatment was replicated three times.

#### Camellia sinensis

The second leaves of tea seedlings were treated with 80 μl per leaf of 1.0 mM lyn3 or an equal amount of buffer in 0.1% Tween 20 via a fine brush. The treatments were repeated once after a 24-h interval. Samples were harvested 1.5, 24, and 48 h after the start of treatment to assess expression levels of related genes and contents of flavonoids separately. Eight replications were carried out.

Twenty-four hours after the final treatment, 20 μl oral secretion(OS) was applied to the mechanical wounds of the second tea leaves to simulate *E. grisescens* feeding [42, 48]. Intact healthy tea plants were used as controls. Samples were harvested at 1.5 and 3 h after the start of treatment to assess phytohormone contents. Each treatment was replicated four times.

JA-treated tea plants were individually sprayed with 8 ml of 600 mg ml^−1^ JA solution, and the control plants were sprayed with 8 ml sodium phosphate buffer (pH = 8). Samples were harvested 12, 24, and 48 h after the final treatment to assess accumulation of flavonoids.

### qPCR analysis

Total RNA was extracted using a TRIzol™ kit (TIANGEN, Beijing, China). Quality and concentration were examined by agarose gel electrophoresis and spectrophotometer analysis. A PrimerScript^®^ RT Reagent Kit (Takara, Dalian, China) was used to synthesize first-strand cDNA from total RNA. The primers of reference and JA-responsive genes in *Arabidopsis* and flavonoid biosynthesis-related genes in the tea plant came from a previous report [22]. The relative expression levels of the above genes were examined by qPCR using a LightCycler 480 (Roche Diagnostics, Mannheim, Germany).

The accession numbers for the genes detected in the study are as follows: *AtCOI1* (AT2G39940), *AtAOS* (AT5G42650), *AtLOX3* (AT1G17420), *AtOPR3* (AT2G06050), *AtJAZ1* (AT1G19180), *AtTHI* (AT1G72260), *AtVSP1* (AT5G24780), *CsPALa* (KY615669), *CsPALc* (KY615671), *CsC4H* (KY615676), *CsANR* (KY615701), and *CsFLS* (ASU87436).

### Measurement of phytohormones and flavonoids

Well-ground tea leaf (0.1 g) was combined with 0.1 g polyvinylpolypyrrolidone cross-linked (PVPP), then 1 ml ethyl acetate containing internal standards (^2^H_2_-JA, ^2^H_6_-JA-Ile, ^2^H_4_-SA, ^2^H_6_-ABA) was added. After vortexing and centrifugation, the supernatant was transferred to new centrifuge tubes and solvent was removed with a centrifugal evaporator; 75% methanol was then added and the material was transferred to vials. Ultraperformance liquid chromatography–mass spectrometry/mass spectrometry (UPLC–MS/MS) was used to determine the contents of phytohormones [49].

Well-ground tea leaf (0.2 g) was extracted with 80% methanol at room temperature and filtered through a 0.22 μm nylon filter membrane, and the contents of flavonoids were measured by UPLC–MS/MS [41].

Well-ground tea leaf (0.1 g) was extracted with 70% methanol at 70°C for 20 min and filtered through a 0.22 μm nylon filter membrane, then high-performance liquid chromatography (HPLC) was performed to determine the contents of catechins [45].

### Bioassay

The first three leaves of tea seedlings were treated with 80 μl per leaf of 1.0 mM lyn3 or an equal amount of buffer in 0.1% Tween 20 via a fine brush. The treatments were repeated once after a 24-h interval. For the lyn3 plus JA treatment, 24 h after the final lyn3 treatment, 8 ml of 50 mM JA was sprayed onto whole tea seedlings individually. Intact healthy tea plants were used as controls. Two caterpillars that had been starved for 6 h were introduced to the treated leaves and then covered with a fine-mesh sleeve 24 h after the final treatment with lyn3, buffer, or JA. The caterpillars were transferred to a nearby treated leaf on the same branch when the leaf was almost half consumed. Each treatment replicated 48–55 times.

Artificial diet bioassay methods were as described by Yang *et al*. [39]. Three-day-old healthy caterpillars of the same size that had been starved for 6 h were fed with artificial diets containing 0, 1, 2, 4, or 8 μg g^−1^ of astragalin and 3, 6, or 12 mg g^−1^ of EGC, which corresponded to the physiological concentrations in tea plants. Each concentration of each compound was replicated 43–54 times.

## Statistical analysis

All statistical analyses were conducted using SPSS Statistics 20 (IBM Inc., New York, USA). One-way analysis of variance (ANOVA) was used to detect statistical differences among three groups. If the ANOVA analysis was significant (*P* < .05), Tukey’s honestly significant difference (HSD) *post hoc* test was used to detect differences between groups. Student’s *t*-test was used to compare the difference between two treatments.

## Acknowledgements

We gratefully acknowledge Jianying Jin for assistance with the experiments. This work was supported by the National Natural Science Foundation of China (32001922 and 31972280).

## Author contributions

X.S. and S.L. conceived the research and designed the experiments; S.L., M.Y., X.L., Y.X., M.L., and J.Z. performed the experiments; S.L. and X.S. analyzed and interpreted the data; S.L. and X.S. wrote the manuscript with contributions from all authors; X.S. supervised the research.

## Data availability

All data needed to evaluate the conclusions in the paper are present in the paper and/or the Supplementary material. Additional data related to this paper may be requested from the authors.

## Conflict of interest

The authors declare no competing interests.

## Supplementary data

Supplementary data is available at *Horticulture Research* online.

## Supplementary Material

Web_Material_uhab038
