# Supplementary material for: A novel inhibitor of the jasmonic acid signaling pathway represses herbivore resistance in tea plants
Source: Hortic Res. 2022 Jan 19;9:uhab038. doi: 10.1093/hr/uhab038 (PMC8945283; doi:10.1093/hr/uhab038)
Supplement: Web_Material_uhab038 [file web_material_uhab038.zip › Supplemental material 2.docx]

**Supporting Information Appendix**

**A novel inhibitor of the JA signaling pathway** **represses herbivore resistance in tea plants**

Songbo Lin^1,2^, Meng Ye^1,2^, Xiwang Li^1,2^, Yuxian Xing^1,2^, Miaomiao Liu^1,2^, Jin Zhang^1,2^, Xiaoling Sun^1,2 *^

^1^ Tea Research Institute, Chinese Academy of Agricultural Sciences, Hangzhou 310008, Zhejiang, China

^2^ Key Laboratory of Tea Biology and Resources Utilization, Ministry of Agriculture and Rural Affairs, Hangzhou, 310008, Zhejiang, China

Author for correspondence: Xiaoling Sun, Email: [xlsun@mail.tricaas.com](mailto:xlsun@mail.tricaas.com) (XS)


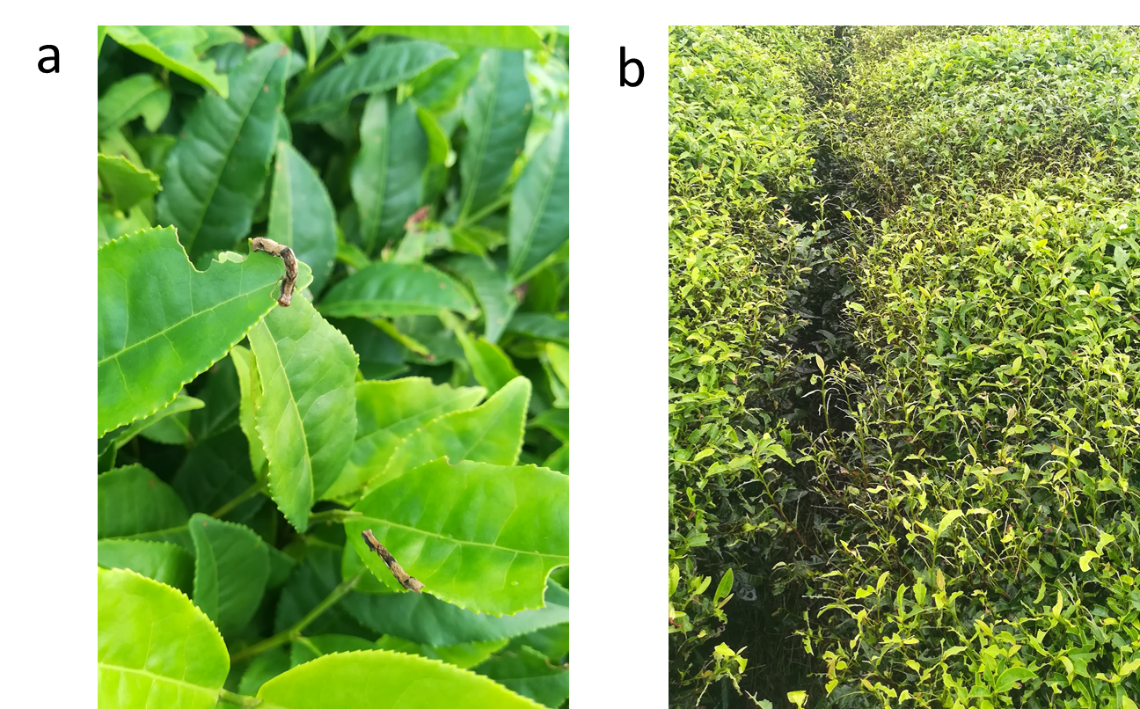


**Figure S1**. *Ectropis grisescens* larvae (a) and field damage (b)


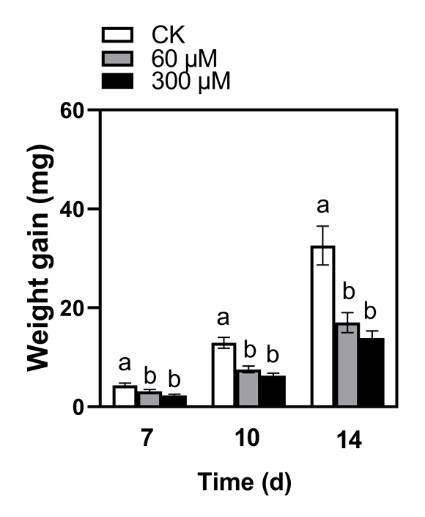


**Figure S2.** Effects of artificial diet supplemented with lyn3 on *E.* *grisescens* larval weight gain (Mean ± SE, *n* = 24-27). Different letters indicate significant differences among treatments (Turkey’s honestly significant difference (HSD) post-hoc test, *P* < 0.05).


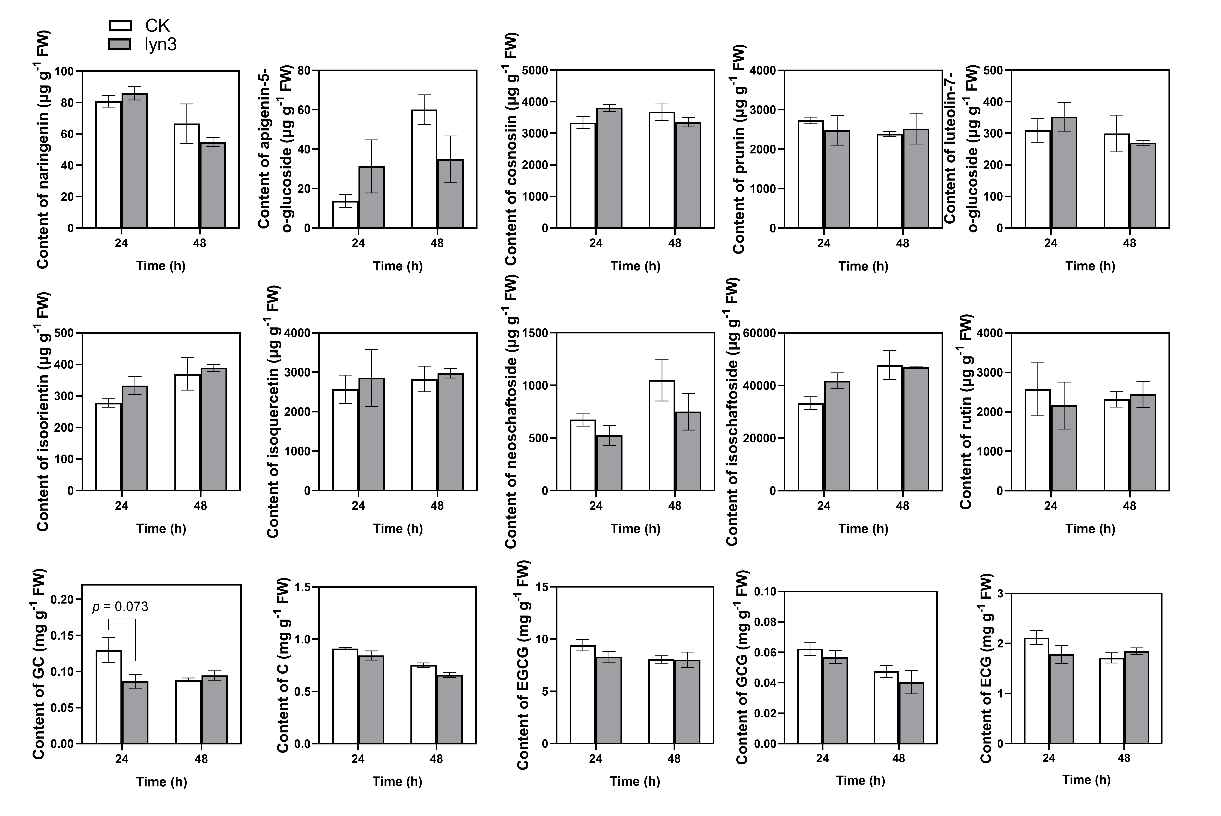


**Figure S3.** Effect of lyn3 on the accumulation of flavonoids in tea plants (Mean ± SE, *n* = 4).


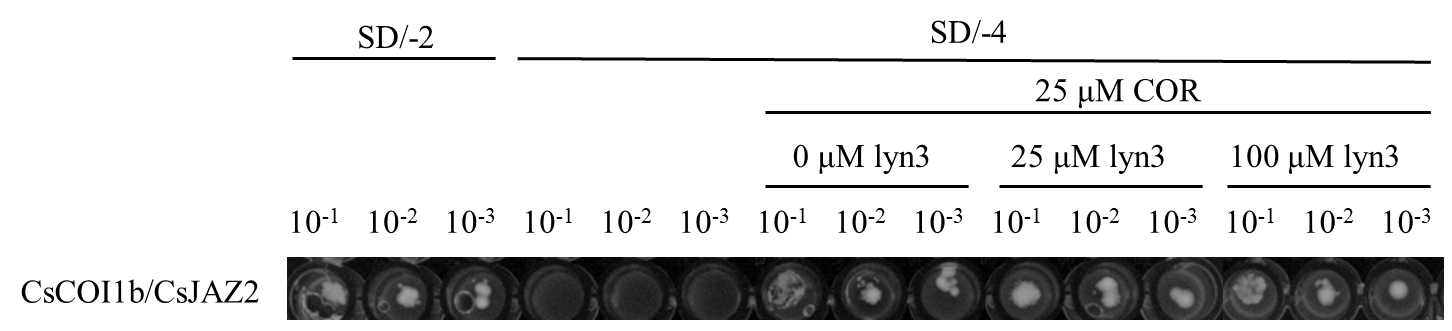


**Figure S4.** Effect of lyn3 on the coronatine-dependent interaction between CsCOI1 and CsJAZ2 in yeast. Growth of yeast cells cotransformed with pGBKT7-CsCOI1b/pGADT7- CsJAZ2 on medium lacking leucine and tryptophan (SD/-2) for transformation control, or on selective medium lacking adenine, histidine, leucine and tryptophan (SD/-4), and supplemented with the indicated molecules.


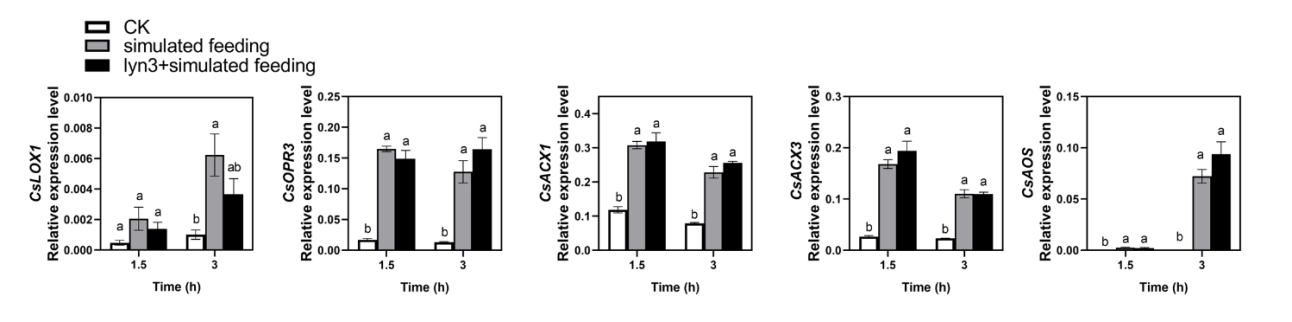


**Figure S5.** Lyn3 had no influence on the *E.* *grisescens* -elicited levels of JA synthesis relative genes (Mean ± SE, *n* = 4). Different letters indicate significant differences among treatments (Turkey’s honestly signiﬁcant difference (HSD) post-hoc test, *P* < 0.05).


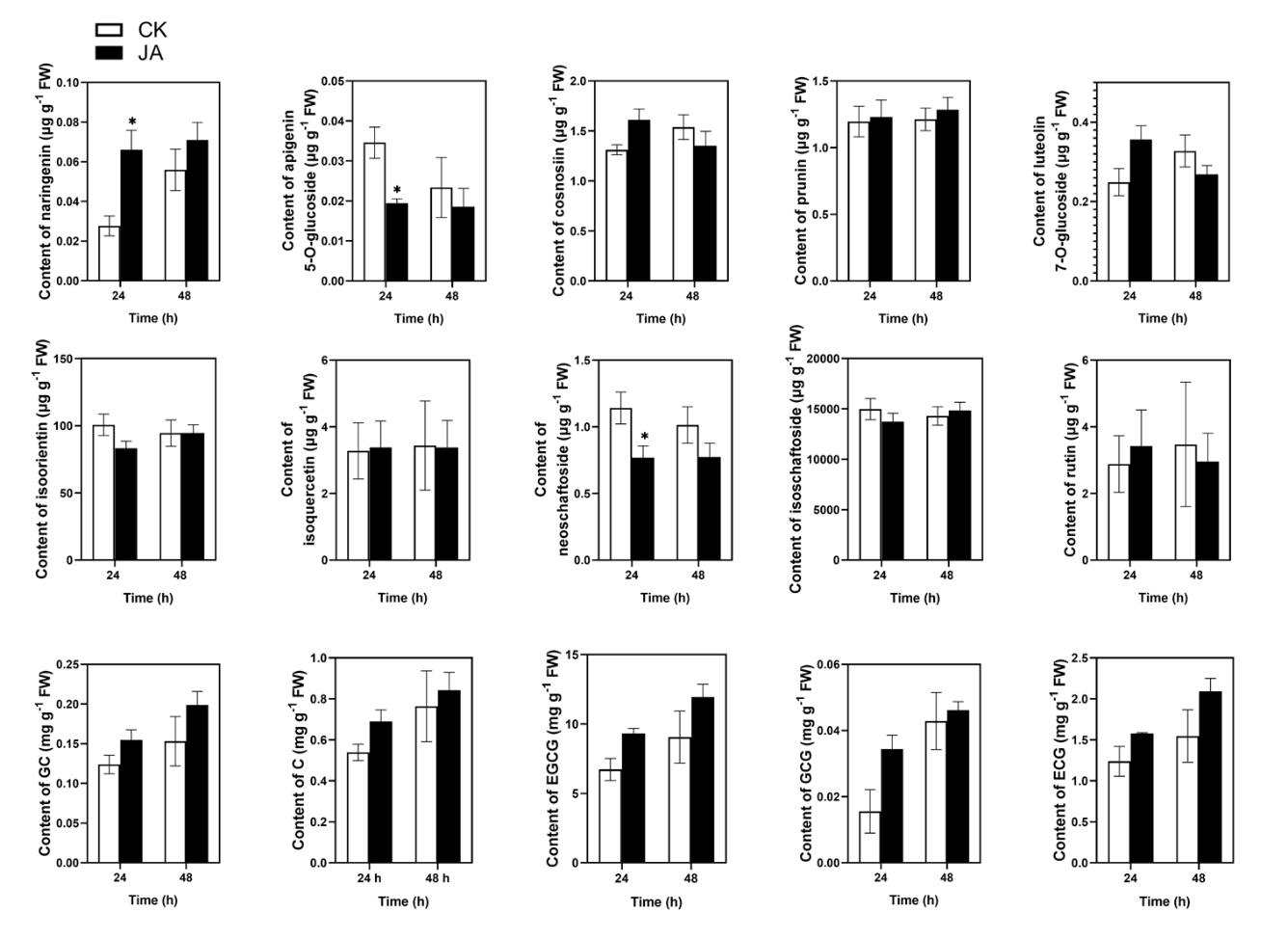


**Figure S6.** Effect of JA on the accumulation of flavonoids in tea plants (Mean ± SE, *n* = 5).
